# A new case of *Echinococcus ortleppi* infection diagnosed by next-generation sequencing in China

**DOI:** 10.3389/fcimb.2025.1719268

**Published:** 2026-01-22

**Authors:** Xishuai Jia, Junhua Tian, Ming Huang, Xuwei Zhou, Jing Liu, Hai Jiang, Kun Li

**Affiliations:** 1 Wuhan Center for Disease Control and Prevention, Wuhan, Hubei, China; 2 Department of Laboratory Medicine, Tongji Hospital, Tongji Medical College, Huazhong University of Science and Technology, Wuhan, Hubei, China; 3 Center for Clinical Laboratory General Hospital of The Yangtze River Shipping Wuhan Brain Hospital, Wuhan, Hubei, China; 4 National Institute for Communicable Disease Control and Prevention, Chinese Center for Disease Control and Prevention, Beijing, China

**Keywords:** China, cystic echinococcosis, *Echinococcus ortleppi*, epidemiology, non-endemic area

## Abstract

Cystic echinococcosis is an important parasitic zoonosis infecting numerous humans with high morbidity and mortality. As one of the etiologic agents, *Echinococcus ortleppi* infection in humans has been very rare. In this study, a 27-year-old man was diagnosed with cystic echinococcosis in Wuhan City of China, a non-endemic area. Next-generation sequencing identified that the etiologic agent was *E. ortleppi.* Its complete mitochondrion sequence (13,600 bp) has 99.92% identity to *E. ortleppi* from cattle in Japan. This is the third reported *E. ortleppi* infection case in China. Although extensive epidemiological investigations were performed, the infection source of this patient is still unclear. It is possible that there exists some hidden or unrecognized route of *E. ortleppi* transmission in China. Further investigation is needed to figure out and eliminate the risk factors.

## Introduction

Cystic echinococcosis is an important parasitic zoonosis infecting humans, wildlife, and livestock. When infecting humans, it can develop asymptomatically for many years and affect multiple organs including livers, lungs, kidneys, and bones (Celik et al., 2025). Cystic echinococcosis is associated with high morbidity and mortality among human populations worldwide, thus posing a great threat to public health. The etiologic agent of cystic echinococcosis, *Echinococcus granulosus sensu lato* (Cestoda: Taeniidae) complex, was practically divided into several species and genotypes based on their phylogenetic positions and the host specificities: *E. granulosus sensu stricto* (genotypes G1–G3, also named sheep strain), *E. equinus* (G4, horse strain), *E. ortleppi* (G5, cattle strain), and *E. canadensis* (G6–G10, cervid strain) (Grenouillet et al., 2014). Of those, *E. ortleppi* has been detected in cattle, sheep, goats, dogs, and camels in many countries and areas (Gholami et al., 2021). However, *E. ortleppi* infection in humans has been very rare. Until today, most human cases were sporadically reported in Europe, Southeast Asia, and South America (Yoshida et al., 2024).

## Case presentation

**Figure 1:**
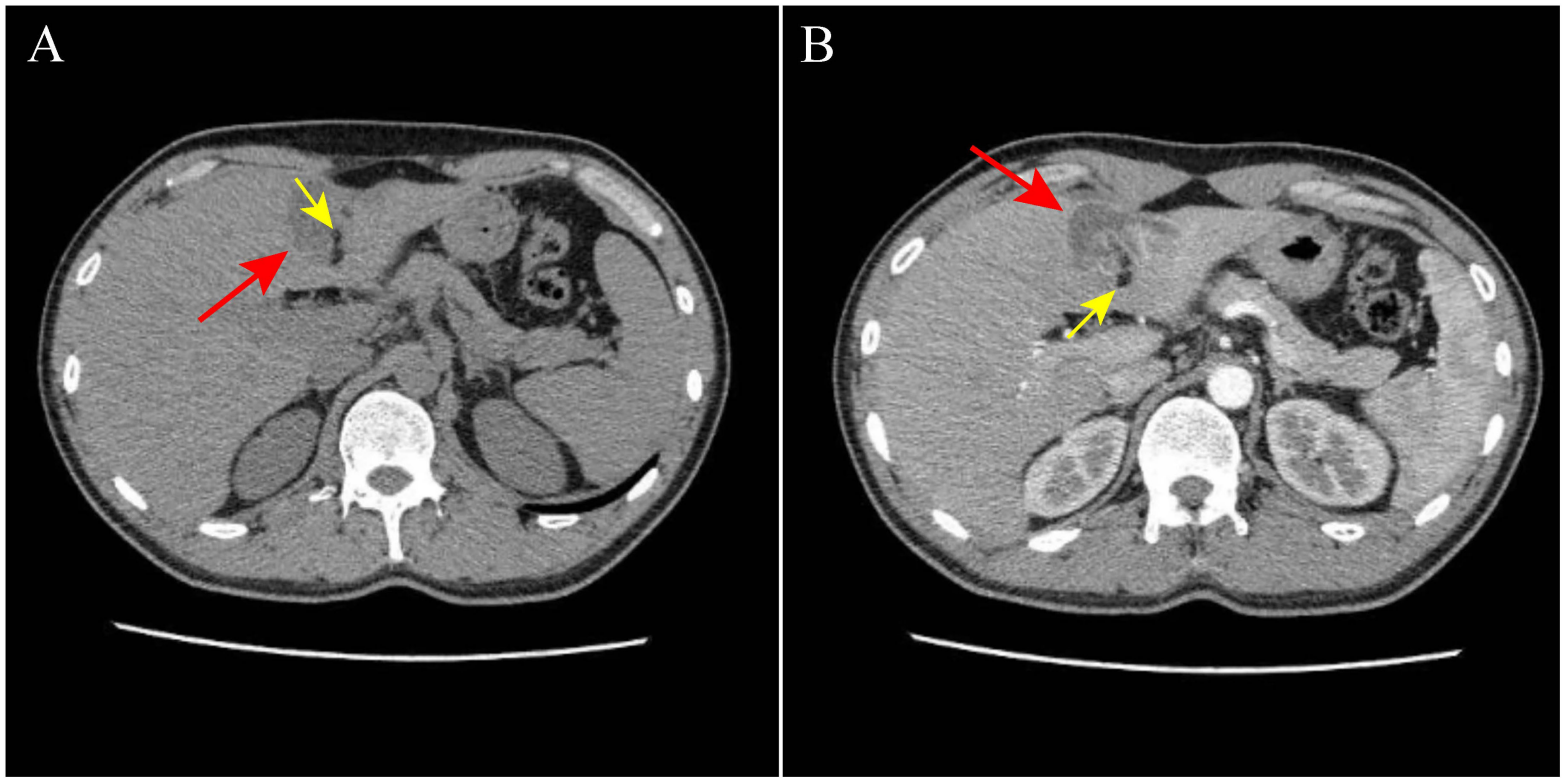
CT on May 27, 2024 (both parts **A** and **B**) showed that segmental enhancements and detached endocysts were in the liver lesions (both pointed by arrows).

**Figure 2:**
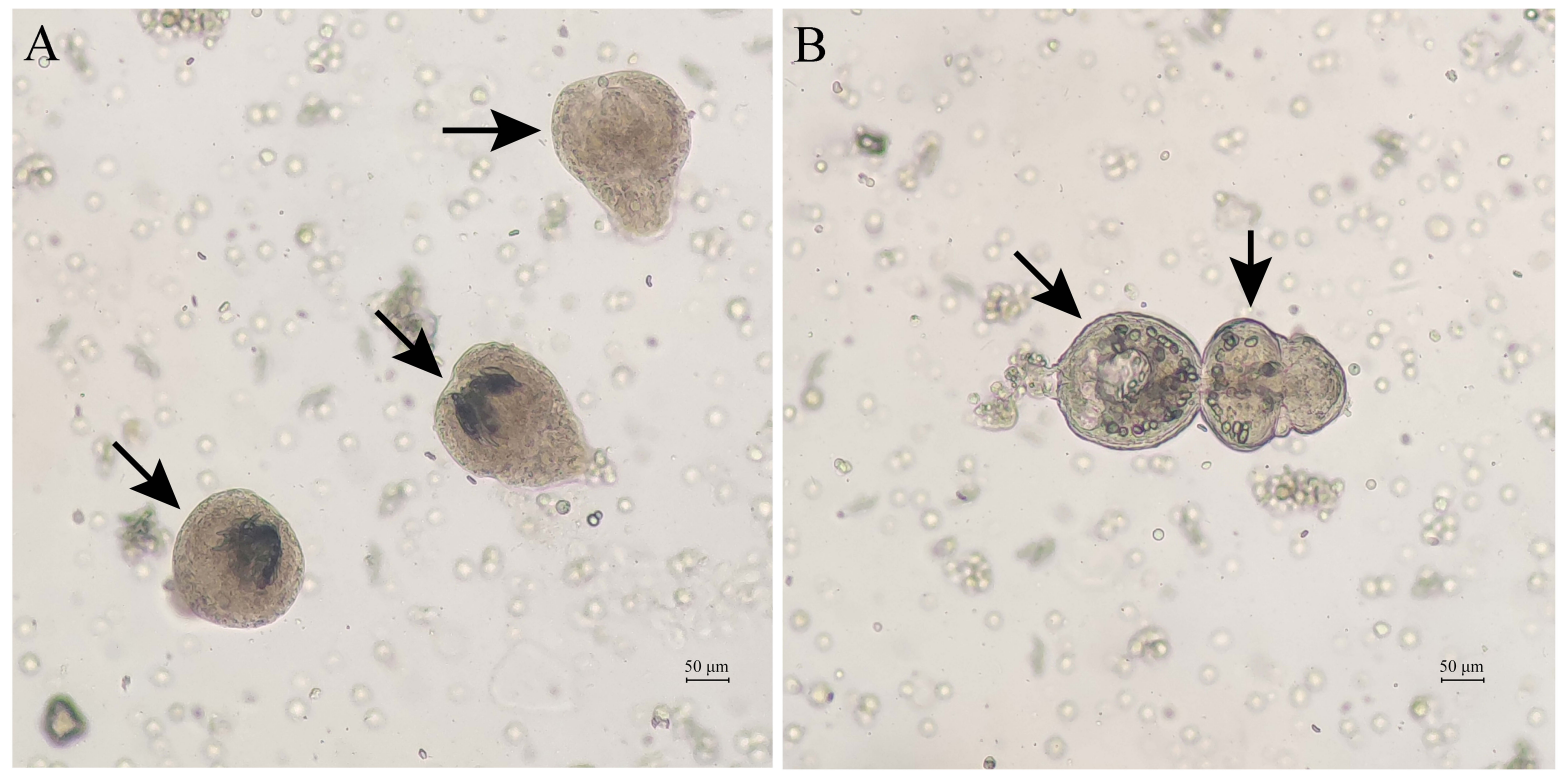
Protoscolices and hydatid cyst shown by the OLYMPUS CX23 microscope (both pointed by arrows). **(A)** Protoscolices. **(B)** Hydatid cyst.

On May 26, 2024, a 27-year-old man presented to Wuhan Fourth Hospital in Hubei Province, central China, complaining intermittent upper abdominal distension and pain for 1 day. The body temperature (37°C), pulse (86/min), and blood pressure (132/81 mmHg) were all normal. The computed tomography (CT) result showed a huge cystic mass beneath the left lobe of the liver. He was then transmitted to Yangtze River Shipping General Hospital for further examination and therapy on the next day (May 27, 2024). The laboratory tests indicated that most results were normal, except for the elevated monocyte count (0.70 * 10^9^/L), decreased aspartate aminotransferase (AST) (12.9 U/L), elevated serum bicarbonate (30.20 mmol/L), and elevated serum magnesium (1.08 mmol/L). The CT result showed that segmental enhancements and detached endocysts were in the liver lesions (**Figure 1**). The boundary was clear and the cyst wall was visible. The size of the lesion was approximately 41 × 28 mm. He was then provisionally diagnosed with cystic echinococcosis. On May 29, 2024, the lesion was excised surgically. A part of the cyst was fixed in 10% formalin solution. The paraffin-embedded sections were stained with hematoxylin–eosin (H&E) and then observed under a microscope at ×40 magnification for pathological diagnosis. Meanwhile, another part of the cyst was preserved in 70% ethanol at 4°C. The suspension liquid of the endocyst rinsed with physiological saline was centrifuged at 1,500 *g* for 5 min at room temperature. After the supernatant was poured out, a wet preparation was examined microscopically for the presence of protoscolex from the sediment. As shown in **Figure 2**, protoscolices and hydatid cyst were clearly observed, supporting the diagnosis of cystic echinococcosis. After surgical resection, the patient was discharged from the hospital, and albendazole was used as an adjunct. In the postoperative follow-up on July 9, 2024, liver dysfunction (elevated alanine aminotransferase [ALT, 57.70 U/L], elevated γ-glutamyl-transferase [γ-GGT, 107.00 U/L], and elevated cholinesterase [12,711 U/L]) was observed, but all results became normal on August 11, 2024.

**Figure 3:**
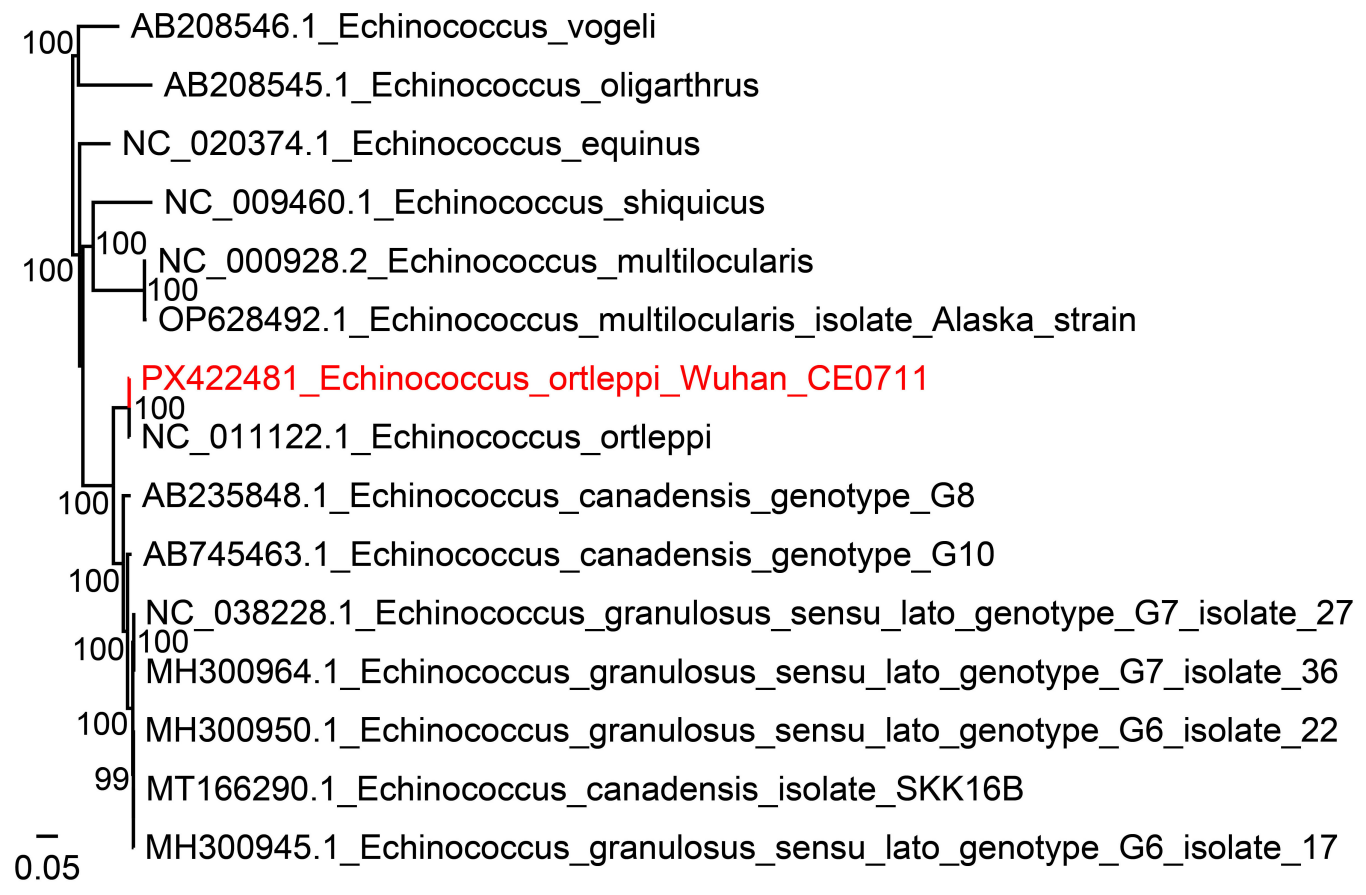
Phylogenetic tree based on the complete mitochondrion sequence of *E. ortleppi* (13,600 bp) and reference sequences. A phylogenetic analysis was performed based on the maximum-likelihood (ML) method implemented in PhyML (v3.0).

For further confirmation and identification of the *Echinococcus* species, DNA was extracted from the hydatid cyst using a DNeasy blood and tissue kit (Qiagen, Germany). The DNA library was prepared using FS Pro DNA Lib Prep Kit V2 following the instruction and then subjected to next-generation sequencing (Wuhan Biobank Co., China). Sequencing was performed on NovaSeq 6000 System (Illumina), and Fastp was used for quality control. The reference sequences used for alignment included *E. ortleppi* (G5) (accession number AB235846) and *E. canadensis* (G6, G7, G8, and G10) (accession numbers AB208063, AB235847, AB235848, and AB745463). A total of 24.3 Gb clean data were recovered, and *Echinococcus* mapped reads (*n* = 858,840) were analyzed against the GenBank Database. Most reads (858,485 of 858,840) were 100% identical to the G5 genotype, indicating that the *Echinococcus* species was *E. ortleppi*. The coverage was 100%, and the average sequencing depth was 5,415.4×. For confirmation, the mitochondrial cox1 and nad1 genes of *E. ortleppi* were amplified by nested PCR (Shi et al., 2019), respectively. The sequences of cox1 (795 bp) gene and nad1 gene (900 bp) both showed 100% identity with *E. ortleppi* (GenBank numbers: KY766908.1, NC_011122.1) in India and Japan. Furthermore, a complete mitochondrion sequence of *E. ortleppi* (13,600 bp) was obtained from the NGS data (GenBank No.: PX422481). Phylogenetic analysis based on the maximum-likelihood method implemented in PhyML (v3.0) indicated that it was closely clustered with *E. ortleppi* (NC_011122.1) from cattle in Japan, with a nucleotide identity of 99.92% (**Figure 3**).

Epidemiological investigation indicated that the patient has been living in Hubei Province, except for a 1-month period of living in Guangdong Province, both of which are not endemic areas of echinococcosis. He has never visited any endemic area of echinococcosis. He has kept a pet cat since 2021 and a pet dog since February 2024. A total of 20 feces samples of the pet dog were collected and examined using the Kato-Katz technique. In addition, 30 human serum samples were collected from his colleagues and neighbors and analyzed using an ELISA kit (Allenda Biotech Co. Ltd., Shanghai, China) to detect *Echinococcus* IgG antibodies. However, no positive result was obtained. He claimed that he frequently ate barbecues during college, but the samples could not be available for detection. Therefore, the infection source of this patient is still unclear.

## Discussion

China has a high prevalence of human echinococcosis. It was estimated that approximately 120, 000 people were infected and 50 million people were under the risk of infection in 2012 (Yu et al., 2020). Most human cases occurred in the endemic areas located in northern and western China. Four *Echinococcus* species have been identified in China. Of those, *E. granulosus* and *E. multilocularis* are the most common species, causing cystic echinococcosis and alveolar echinococcosis, respectively. In contrast, *E. ortleppi* has been considered a rare pathogen in humans in China. Until today, there have been only two formally reported human cases of *E. ortleppi* infection. The first case was discovered in 2019 in Guangxi Zhuang Autonomous Region, southwest China (Shi et al., 2019), while the second was reported in 2021 in Guizhou Province, southwest China (Wang et al., 2021). This may be the third case of *E. ortleppi* infection in China. Although the symptoms of the current patient were similar to those of the previous two patients (pain and distension in the upper abdomen without other symptoms), the lesion site was in the left lobe of the liver, which was different from the previous patients. In addition, the nad1 and cox1 genes were obviously different from the available sequences of patients (98.30% and 99.57% nucleotide identity for the Guangxi patient and 99.23% and 99.66% for the Guizhou patient, respectively), hinting that they may have different origins.

Detecting *E. ortleppi* in a non-endemic region may suggest a remarkable risk to public health. First, it may pose diagnostic challenges of identifying echinococcosis in non-endemic areas. For most hospitals in non-endemic areas, serological assays are not available, while clinical judgments solely based on imaging examinations may lead to misdiagnosis or delayed diagnosis. For early discovery and to reduce the risk of diagnosis, regional surveillance programs targeting mammal hosts (cattle, dogs, pigs, etc.) and patients with suspicious symptoms may be helpful. Second, there may exist some undiscovered endemic areas or neglected transmission routes. According to the epidemiological investigation, the infection source of this patient was still not determined. For almost all the time in life, he has been living in Hubei Province, central China. Therefore, it is quite possible that he was infected in Hubei Province. It is possible that there exists some hidden or unrecognized route of *E. ortleppi* transmission (for example, eating uncooked food) in central China. Besides that, this result may also expand the endemic area of echinococcosis in China. Notably, although *E. ortleppi* was rarely reported in China, lots of DNA sequences of *E. ortleppi* have been uploaded to the GenBank Database (OP471634.1-OP471636.1 [from pig livers in Yunnan Province], OR604367.1 [from canine fecal sample in Tibet], OP471630.1 [from yak liver in Yunnan Province], etc.), indicating that its distribution in China is larger than previously recognized. Its hosts include dogs, cows, pigs, and yaks. With the development of modern food distribution and logistics networks in China, livestock products may be transported over long distances from their original place. Although humans are not infected through the consumption of meat itself, indirect exposure to *Echinococcus* eggs via contaminated environments, food handling processes, or contact with infected canids associated with livestock production cannot be excluded. Therefore, the possibility of infection through such indirect pathways warrants further investigation.

Our work also has some limitations. Although we performed some epidemiological investigation, the depth and scope of investigation are not quite enough—for example, the local slaughterhouses, stray dogs, and livestock sources have not been surveyed. In addition, the Kato-Katz method may not be sensitive enough for *Echinococcus* detection in canids. In future studies, further investigation is needed to figure out and eliminate the risk factors.

## Data Availability

The datasets presented in this study can be found in online repositories. The names of the repository/repositories and accession number(s) can be found in the article/supplementary material.
